# Land Use Change Effect on Soil Carbon Stock and Selected Soil Properties in Gobu Sayyo District, Western Ethiopia

**DOI:** 10.1155/tswj/4726114

**Published:** 2025-08-06

**Authors:** Deginet Wako, Mengistu Welemariam, Getahun Kitila

**Affiliations:** ^1^Department of Soil Resources and Watershed Management, Wollega University, Shambu, Oromia, Ethiopia; ^2^Department of Soil Resources and Watershed Management, Aksum University, Shire, Tigray, Ethiopia

**Keywords:** deforestation, land degradation, land use change, soil carbon stock, soil properties

## Abstract

Land use change is one of the major factors affecting soil degradation. Growing population pressure has increased land use change with more negative effects on soil carbon storage and overall soil properties. The objective of this study was to assess the effect of land use changes on soil organic carbon (SOC) stock and selected soil physicochemical properties in Gobu Sayyo, Western Ethiopia. Soil samples were collected from three adjacent land uses, that is, forest land, grazing land, and cultivated land, at 0–20 and 20–40 cm soil depths. A total of 36 composite soil samples were collected, and the major soil properties and SOC storage of the area were analyzed and computed based on their standard procedures. SOC stock was significantly (*p* < 0.05) higher (43.09–81.86 t ha^−1^) in forest land and was significantly lower (38.08–43.09 t ha^−1^) in cultivated land at the top 20 cm. SOC stock decreased with depth in all land uses. Changes in land use and soil depth affected the physical and chemical properties of soil. The physical soil property such as bulk density (BD) was higher (1.62 g cm^−3^) in the cultivated land, whereas the lowest (1.08 g cm^−3^) was recorded in the forest at 0–20 cm depth. Comparatively, the moisture content was higher (25.89%) under forest land at the depth of 20–40 cm and was lower (11.22%) under cultivated land. The chemical soil properties like exchangeable Ca^2+^, Mg^2+^, and K^+^ were higher in forest lands. Organic carbon, available phosphorus (AvP), total nitrogen (TN), exchangeable calcium (ex.Ca^2+^), exchangeable magnesium (ex.Mg^2+^), exchangeable potassium (ex.K^+^), and cation exchange capacity (CEC) were lower under cultivated lands. pH increased with depth and was higher under forest land and lower under cultivated land. Soils of the study area are in general acidic to slightly acid, with pH values ranging from 4.6 to 6.02. The pH, SOC, TN, AvP, and CEC were higher under forest land compared to cultivated and grazing lands. It can be concluded that SOC stocks and the physical and chemical properties were affected by land use change and depth. Therefore, reducing the intensity of cultivation, adopting integrated soil fertility management, and maintaining forest land must be practiced to save the soil of the area from degradation.

## 1. Introduction

Organic matter (OM) is a key component of the carbon (C) cycle and represents the largest terrestrial repository of C globally [1]. Hence, the capability of the soil to store and preserve OM has received much attention, to develop strategies to manage soils to increase their C storage and reduce atmospheric carbon dioxide [2]. Vegetation plays a significant role in the formation of soil organic matter (SOM) and influences fundamental soil-forming processes such as aggregation or podzolization [3–5]. Anthropogenic activity such as deforestation, agricultural expansion/intensification, development of infrastructures, and land use changes with invasives adversely affected the regeneration pattern and caused a reduction in biodiversity as well as C stock [6–9]. Due to the abovementioned disturbances, land use systems were adversely affected in terms of microbial activities, nutrient cycling, and reduced SOM and productivity [10, 11].

SOM plays a key role in ensuring the long-term conservation of soil resources [12]. Adequate levels of OM are essential to maintain or improve soil physical properties like soil porosity, infiltration capacity, moisture retention, and resistance to water and wind erosion and chemical properties like soil reaction and nutrient availability [12].

Soils store two or three times more C than that exists in the atmosphere as CO_2_ and 2.5 to 3 times as much as that stored in plants [13]. Man-induced alterations affect not only the total C content of soils but also its distribution among the various pools [1], causing changes in the size distribution and stability of aggregates, as well as other soil properties [14]. Changes in land use and management have profound effects on the quantity and dynamics of SOM and, in turn, on the soil ecosystem functions [1]. It has contributed to soil degradation and soil loss, leading to a decrease in soil C storage [15]. Land use change due to deforestation in the tropics was the major contributor to CO_2_ emissions in the 1990s [16]. Particularly, it is well studied that converting natural forests into agricultural fields [17] leads to a decline in OM, shrub lands [18], grass lands [5, 9], and other land use [6].

The pressures of the human population on land resources have increased land use change with more negative effects on soil properties [19]. Land use transformation can affect soil physical and chemical properties. Soil nutrient availability is greater in forest land than in the cropland and pastures that replace them [20]. Forest conversion to cropland can also decrease soil properties such as soil texture, porosity, phosphorus (P) content, and cation exchange capacity (CEC) but increase soil bulk density (BD) [21].

Most of the land use changes in Ethiopia are due to direct and indirect human activities. These include overpopulation, deforestation, and urbanization, which led to the loss of natural resources [22]. Soil resources are prone to degradation due to misuse and mismanagement [23]. Besides, intensive agriculture and long-term exploitative farm practices have led to continuous depletion of natural vegetation cover and overutilization of land resources. Lack of agricultural inputs, traditional farming methods, and overgrazing aggravated the degradation of soil physical and chemical properties in Ethiopia [21]. On the other hand, integrated soil fertility management maintains the physical and chemical properties of soils.

Ethiopia was once covered with greater than 35% forest cover in the 1940s. However, due to the increased human and livestock population, a large part of the forest is converted to farmlands and grazing lands. For sustainable utilization of natural resources, assessment of man-induced land use change is therefore very important. Many studies have been conducted on the effect of land cover–land use change on soil properties in the country [22]. However, there is limited information on the effect of land use change on SOC storage and soil properties in the western part of the country including Gobu Sayyo district.

Study on the effects of land use changes could be effective in recognizing suitable management practices that help in declining atmospheric CO_2_ concentration and soil quality (Gobu Sayyo Agriculture & Natural Resources, 2013).

Gobu Sayyo district in Western Ethiopia has a large population size and a shortage of farmland. The rapidly increasing population pressure in the district has led to vast changes in land use patterns mainly caused by increasing agricultural production at the expense of forest and grazing lands (Gobu Sayyo Wereda Agricultural Office, 2009). Despite this problem, no research was done in the district on the effect of land use change on soil C stock and soil properties. Thus, this study was undertaken to determine the effect of land use changes on soil organic carbon (SOC) stock and selected soil properties in the district with the assumption that land use change will bring a significant effect on soil C stock and selected soil properties.

## 2. Materials and Methods

### 2.1. Description of the Study Area

**Figure 1:**
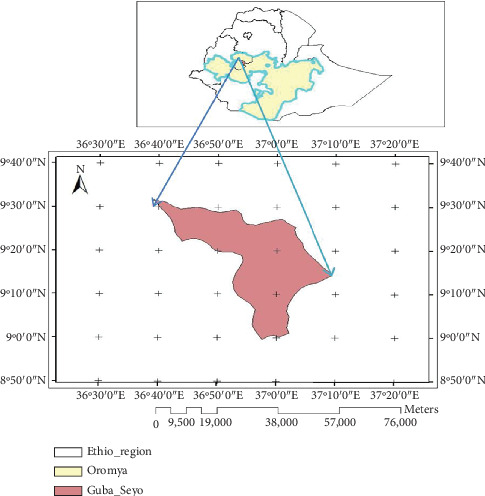
Map of the study area.

Gobu Sayyo is the district found in East Wollega Zone, Western Ethiopia, at about 261 km from Addis Ababa, the capital city of Ethiopia. It is located at 8°50⁣^″^0⁣′ N–9°40⁣^″^0⁣′ N and 36°30⁣^″^0⁣′ E–37°20⁣^″^0⁣′ E direction (Figure 1). Most of the land has an elevation of 1500–1960 m above sea level and is characterized by subtropical climatic conditions, with a mean annual temperature ranging 13°C–27°C and mean annual rainfall of 770–1657 mm (Gobu Sayyo District Office, 2018). According to the World Reference Base soil classification, the dominant soil types in the area include Alfisol and Nitisol [24]. The area has rugged topography. The population size of the district is about 57,455, where 27,268 (47.46%) are males, whereas about 30,186 (52.53%) are females, as reported by Gobu Sayyo Health Center (GSHC, 2019). Mixed livestock–crop production is the major farming system in the area.

Gobu Sayyo district has good vegetation cover than the other neighboring districts. There is a natural forest having a total area of about 1381 ha. However, there is a very serious deforestation. According to Gobu Sayyo Woreda Agricultural Office (GSWAO), out of the total land of the district, the proximate areal coverage of land used for crop cultivations is 21,640 ha, while 6907 ha is pasture or grazing land (GSWAO, 2018).

### 2.2. Sampling Technique, Sample Size, and Preparation

Gobu Sayyo district has eight kebeles. From a total of eight kebeles in the district, two kebeles (Gambella Tare and Ago Sombo) were selected purposely for the study because of their representativeness in terms of the extensive land use changes that happened in the district. From the two kebeles, soil samples were collected from adjacent forest land, cultivated land, and grazing land at two soil depths (0–20 cm) and (20–40 cm) in three replicates. A total of 36 composite soil samples were collected from all the land uses in 2019.

The composite soil samples from representative sites of each land use were collected by auger (for disturbed soil sample) and by core sampler (for BD). Soil samples were packed in a plastic bowl and transported to the soil testing center for further analysis. In the laboratory, samples were labeled, air dried, cleaned from contaminants and plant debris, ground by mortar and pestle, and finally sieved with a 2-mm sieve for analysis. Analysis of soil samples was carried out at Bako Research Center based on their standard laboratory procedures.

### 2.3. Soil Lab Analysis

After soil samples were well prepared, BD was determined by dividing dry soil by its volume after drying to constant weight at 105°C for 24 h. The pH was determined by potentiometry on soil:water suspensions (soil:water ratio 1:2.5) [25]. The CEC was determined by NH_4_Ac extraction followed by H_2_SO_4_ titration at pH 7 [26]. The exchangeable Ca and Mg were determined by NH_4_ acetate extraction followed by EDTA titration. Exchangeable K was determined by NH_4_Ac extraction followed by a flame photometer [27]. Soil texture was analyzed by the hydrometer method [28]. Soil moisture content was determined by the gravimetric method [29]. Total N was determined by Kjeldahl [30]. The percent of organic C was analyzed by wet oxidation with dichromate [31]. Available P was determined by Bray II, 1945, as the soil is acidic.

### 2.4. Estimation of SOC Stocks

SOC stock was estimated up to the depth of 40 cm. The SOC stock was calculated as indicated in the following equation:

 $$\begin{array}{c} \text{SOCst}=\text{SOC}\%/100*\text{BD}*D \end{array}$$

where SOC is the soil organic carbon (percentage) of a given soil depth, SOCst is the soil organic carbon stock (kg C ha^−1^), BD is the soil mass per sample volume (kg m^−3^), and *D* is the depth of soil in meter.

### 2.5. Statistical Analysis

The soil property data generated through laboratory analysis were subjected to a two-way analysis of variance (ANOVA) using the general linear model of SAS Version 9.2 to detect whether differences in soil attributes existed between the land use and soil depth using the general linear model procedure of the Statistical Analysis System [32]. The least significant difference (LSD) test was employed for mean separation for the soil properties that were found to be significantly different in statistical terms.

## 3. Results and Discussion

### 3.1. Effect of Land Use Change on SOC Stock

The ANOVA results (Table 1) indicated that the SOC stock was significantly (*p* < 0.05) affected by land use types and soil depth. The mean value of C stock under cultivated soil, forest soil, and grazing soil at the depth of 0–20 cm was 43.09, 81.86, and 57.71 t ha^−1^, respectively, whereas, at a depth of 20–40 cm, the value of SOC stock under cultivated soil, forest soil, and grazing soil was 38.08, 70.31, and 44.54 t ha^−1^, respectively. In the highly dissected landscape, bioclimatic conditions change rapidly and could vary within short distances, resulting in a pronounced heterogeneity of soils and their physical, chemical [3, 17], and biological characteristics [6]. This could directly affect the vegetation types and their functional traits [33, 34].

Forest land had the highest SOC stock, and cultivated land had the lowest value. Next to the forest, grazing land had the highest SOC stock. OM input and turnover rates are the drivers for the soils' C stock. Tree species strongly influence the forest floor in terms of C stock [35]. The declination of SOC stock in cultivated land could be due to vegetation loss and unsustainable soil management. The present finding agreed with [36, 37] who reported that the SOC loss in cultivated soil could be due to reduced OM input, as well as due to reduced physical protection of soil from erosion and the increased decomposition rate as a consequence of tillage.

Numerous studies reported decreasing SOC stocks after a land use change from natural or seminatural ecosystems (forest land and grassland) to cropland and a cultivation induced SOC decline of about 20%–60% when forest land and grassland were converted to cropland [38–40]. The C stocks increased by 20%–50% after land use changes from cropland to grassland or forest land [36]. The mean value of SOC stock of all land uses decreased significantly as depth increases.

### 3.2. Effect of Land Use Change on Selected Soil Physical and Chemical Properties

#### 3.2.1. Soil Physical Properties

##### 3.2.1.1. Soil Texture

As the result in Table 2 revealed, the mean value of the percentage of sand in cultivated land was 61% and that of forest land was found to be 52% at a depth of 0–20 cm. The highest percent of sand was found in cultivated land, while the lowest was found in grazing land (50%). At the depth of 20–40 cm, the percent of sand under cultivated land was 58%, that of forest land was 45%, and that of grazing land was 54%.

The mean values of silt at a depth of 0–20 cm under cultivated, grazing, and forest lands were 33.5%, 38%, and 43.5%, respectively, while at the depth of 20–40 cm, they were 30.5%, 37%, and 44%, respectively. The percentage value of clay soil at a depth of 0–20 cm under cultivated, grazing, and forest lands was 5.5%, 10%, and 6.5%, respectively, while at a depth of 20–40 cm, the clay content under cultivated, grazing, and forest lands was 11.5%, 9%, and 11%, respectively. The textural class for cultivated land at depth of 0–20 and 20–40 cm was sandy clay loam and sandy loam, respectively. For grazing land, the textural class at both depths (0–20 and 20–40 cm) was loam soil. The textural class at depths of 0–20 and 20–40 cm was sandy loam. Unlike sandy soil, clay soil was higher in forest land than in both cultivated and grazing lands. Despite this, the slightly higher silt content was observed in grazing land, while the lower was in cultivated land, which agrees with research [41] that reported the highest silt content was observed in forest land and the lower was in cultivated land.

In this finding, the highest sand contents (61%) in the cultivated land were in contrast to the result of [42] who said that the highest sand and silt were found in forest land in Warandhab area, Horo Guduru Wollega Zone, Oromia, Ethiopia. However, the highest silt contents (44%) at the depth of 20–40 cm in forest soil land agree with this finding. The highest sand content in cultivated land is perhaps due to a high amount of rainfall in the area that washed away the finer soil particles (clay) leaving behind the sand fractions [43].

##### 3.2.1.2. BD

As indicated in Table 3, the BD values were slightly different for the land use types. The mean value of BD under forest, cultivated, and grazing land at the depth of 0–20 cm was 1.08, 1.62, and 1.63 g cm^−3^, respectively, whereas, at the depth of 20–40 cm, it was 1.26, 1.60, and 1.31 g cm^−3^, respectively. Comparatively, cultivated land had significantly (*p* < 0.05) higher (1.62 g cm^−3^) BD at the depth of 0–20 cm. This might be due to the compaction of the topsoil by cultivation or due to low OM, while forest land had the lowest (1.08 g cm^−3^) BD, which could be due to the presence of high OM. An increase in root penetration and biological activity might have decreased BD in forest soils. The result indicated that surface soil had significantly lower BD than subsurface soil. This could be due to the presence of higher OM in surface soil (Table 3). This agreed with the findings of [44] where the topsoil had less BD than the subsurface soil. This could also be due to overgrazing in the grazing land and intensive agricultural practices in the cultivated land.

##### 3.2.1.3. Soil Moisture

As shown in Table 3, the moisture content (percentage) of the surface soils (0–20 cm) of the study area, forest soil, cultivated land, and grazing land has the mean value 20.6, 10.84, and 14.76, respectively, and at the depth of 20–40 cm, the mean values of moisture in forest soil, cultivated soil, and grazing soil were 25.85, 10.86, and 15.12, respectively. The surface (0–20 cm) soil had lower moisture content than that of the lower depth (20–40 cm). This might be due to solar heating at the topsoil compared to the subsurface soil. Forest land had significantly higher moisture content, whereas cultivated land had the lowest moisture content; this implies that the soil under forest was covered by vegetation, which results in low evaporation; water stays in the soil. This result agreed with [45, 46] who reported that soil protected by the superficial layer of OM improves the capture and the use of rainfall through increased water absorption and infiltration and decreased evaporation from the soil surface. This leads to reduced runoff and soil erosion with higher soil moisture throughout the season compared to the disturbed soils left unprotected [47].

#### 3.2.2. Soil Chemical Properties

##### 3.2.2.1. Soil pH

Soil pH was found to be significantly (*p* < 0.05) affected by land use change and soil depth. Forest soil has the highest mean value of pH (6.20) at the depth of 20–40 cm, whereas it was significantly lower (4.63) in cultivated lands at the depth of 0–20 cm (Table 4). The lower pH at the cultivated land when compared with forest land and grazing lands implies that the depletion of basic cations in crop harvest and the continuous use of ammonium-based fertilizers such as diammonium phosphate (NH_4_)_2_HPO_4_ in cultivated lands make the soil acidic. The oxidation of these fertilizers by soil microorganisms produces inorganic acids. This acid releases H^+^ to the soil solution that in turn lowers the pH of the soil. In general, as the depth increases, the pH value decreases (acidity increase). This is because of the larger OM content observed in the surface soils across the land uses, and the humified OM can bind tightly with Al^3+^ and reduce their activity in the soil solution, which raises the soil pH. The present study is in line with a study by [48] who reported that, at low soil pH, exchangeable base cations become less available while others, such as aluminum, become more available and can reach toxic levels. The basic cations such as Ca^2+^ and Mg^2+^ also showed a decreasing trend with increasing depth. The adsorption of these cations on the colloidal complex gives replacement of H^+^ and Al^+3^ which lowers the percentage of acid saturation and increases the soil pH.

##### 3.2.2.2. SOC

As presented in (Table 4), SOC was significantly (*p* < 0.05) affected by land use change and soil depth. In both soil depths (0–20 and 20–40 cm), SOC was lower in cultivated fields compared to other land uses. The analysis of the effect of soil depth showed the highest SOC (3.79%) under forest soils at 0–20 cm, while the lowest SOC (1.19%) was recorded in cultivated soils at the depth of 20–40 cm. This agreed with [11] and Mengistu and Fassil [9] who reported that SOC storage and soil nutrient availability are greater in forest land than in the cropland and pastures that replace them. Extensive deforestation and the conversion of natural forests into agricultural lands in the Ethiopia ecosystem led to a significant decline in OM levels. As [49] reported, the conversion of forest land into grazing land and cultivated land led to a large decrease of OM at Chemo Watershed Ethiopia. Most cultivated soils of Ethiopia are poor in OM contents due to the low amount of organic materials applied to the soil and complete removal of the biomass from the field [50] and due to steep relief conditions, intensive cultivation, and excessive erosion hazards [51]. In agreement with this, all agricultural fields in the study area had low organic C content according to the classification presented in [52]. It was also stated [53] that an OM content of less than 2% is an indication of soil degradation for tropical soils involving a high risk of soil erosion.

##### 3.2.2.3. Total Nitrogen (N)

The result presented in Table 4 showed there were significant (*p* < 0.05) variations in total N among the land uses and soil depth. The mean value of total N at the depth of 0–20 cm for cultivated land, forest land, and grazing land was 0.11%, 0.33%, and 0.20%, respectively, while, at the depth of 20–40 cm, it is 0.10%, 0.27%, and 0.15%, respectively. Total N increased in the order of cultivated land < grazing land < forest land in the study area. The present finding agreed with [23], who reported that the average total N increased from cultivated to grazing and forest land soils, which again declined with increasing depth from surface to subsurface soils. Since forest land had higher OM, higher TN was recorded in forest land than cultivated and grazing lands at both depths (Table 4). This agreed with the findings of [54] where the total N was measured higher in soils with high OM.

Generally, cultivated soils had significantly lower TN at all depths compared to grasslands and forest lands, which indicated that continuous cultivation ultimately reduced the total N contents in the soil. Due to land use shifts from forest land to cultivated land, the TN content declined, and it also declined with increasing soil depth. This finding agreed with the findings of [23] in Senbat subwatershed, Western Ethiopia, where average TN increased from cultivated to grazing and forest soils. The minimum change of TN under cultivated land compared to forest land and grazing land showed that fertilizer applications may not have replaced the total N lost due to harvest removal, leaching, and humus losses associated with cultivation [55]. Continuous cultivation could have also aggravated OC oxidation and loss of N in cultivated fields, resulting in the lowest TN content [12].

##### 3.2.2.4. Available P

As shown in Table 4, the available P content (mg kg^−1^) of the surface soils (0–20 cm) in forest soil, cultivated soil, and grazing soil had mean values of 27.83, 12.63, and 4.17, respectively, and at the depth of 20–40 cm, the mean values of available P in forest soil, cultivated soil, and grazing land soil are 24.53, 10.43, and 2.43, respectively. The surface (0–20 cm) soil has significantly (*p* < 0.05) higher available P content than that of the lower depth. The available P content of soils significantly declined due to the conversion of natural forests into grazing lands and farmland. The high content of available P in forest land could be due to the high content of soil OM, resulting in the release of organic P, thereby enhancing available P under forest land. Similarly, this result is in agreement with the findings of [56] who reported that the available P was high in forest land compared to pasture land and cultivated land at the 0–30 cm soil depth.

The higher available P content at both depths under forest land is likely the consequence of the long-term litter accumulation and the associated increase in microbial activity. The results were in agreement with those of [57] who reported that OM influenced P in soil solution by complexing P from adsorption sites in ligand exchange and increasing the mobility of inorganic P, particularly in acid soils, by decreasing the chemical activity of iron and aluminum. The lack of available P in the soils limits the growth of both cultivated and uncultivated plants. Cultivated land should be supplied with inorganic fertilizer to increase the concentration of P in the soil solution that is required by crops. P is involved in several key plant functions, including energy transfer [58].

High availability of P existed in forest land and the lowest was in cultivated land. This might be the pool of available P could be trees in the forest land with abundant microorganisms that return via litterfall to the soil [59]. In agricultural land, available P was reduced most probably due to a decline in both %SOC, CEC, and soil acidification [60, 61]. This finding also agrees with the result of [62] who reported alteration of forest land to agricultural land decreased availability of P in Western Ethiopia.

In contrast to this finding, Reference [42] found higher available P in the soil of cultivated land as the result of crop residue left on cultivated land and later plowed in properly. In forest land, fallen vegetation cover and natural pruning of trees take time to return to the soil to decompose and increase SOM content, which leads to increases in available P content. Consequently, available P content was significantly (*p* < 0.05) different among different land use systems [63].

##### 3.2.2.5. Exchangeable Cations (K, Ca, and Mg)

The exchangeable K of soil under the study area was significantly (*p* < 0.05) affected by land use type and depth of soil (Table 4). Exchangeable K content at the depth of 0–20 cm in forest land, cultivated land, and grazing land was 1.51, 1.04, and 1.13 Cmol(+) kg^−1^, respectively. At the depth of 20–40 cm, exchangeable K of forest land, cultivated land, and grazing land is 0.79, 0.88, and 0.44 Cmol(+) kg^−1^. Soils of forest land had the highest exchangeable K (1.51 Cmol(+) kg^−1^) content compared to soils of cultivated and grazing lands at the surface soil (depth of 0–20 cm) whereas cultivated land had the least exchangeable K content at this depth, and exchangeable K content of the soil declined with an increase in depth. This result is supported by previous findings that indicate the intensity of weathering, cultivation, and the use of acid-forming inorganic fertilizers affect the distribution of K in the soil system and enhance its depletion [12]. Similarly, Reference [64] reported that the exchangeable K of soil is higher in forest land than in cultivated and grazing lands.

The ANOVA results indicated that the exchangeable Ca was significantly (*p* < 0.05) affected by land use types and soil depth. The presence of such significant variation in exchangeable Ca could be due to different management practices. At the depth of 0–20 cm, the mean values of exchangeable Ca under forest, cultivated, and grazing lands were 23.30, 9.27, and 14.43 Cmol(+) kg^−1^, respectively. However, at the depth of 20–40 cm, its mean value under forest, cultivated, and grazing lands was 12.27, 6.87, and 8.17 Cmol(+) kg^−1^, respectively. This implies that the exchangeable Ca was higher at the surface soil depth than that of the subsurface soil depth. This could be due to the abundance of animal and plant residues at the surface layer of soil compared to the subsurface soil layer. This is agreed with the findings of [63] who in their results revealed that the exchangeable Ca content of the soil was higher on the surface soil layer than the subsurface soil layer due to the association of biological accumulation with biological activity and accumulation from plant residues. In contrast, Reference [65] reported that the exchangeable Ca was increasing with increasing soil depth since it is susceptible and the possibility of being easily leached downward by runoff and water percolation.

The ANOVA results indicated the exchangeable Mg was significantly (*p* < 0.05) affected by land use types and by soil depth. The exchangeable Mg concentrations followed a similar trend as that of Ca under the land uses. The mean value of exchangeable Mg at a depth of 0–20 cm under forest, cultivated, and grazing land uses was 12.70, 5.83, and 6.77 Cmol(+) kg^−1^, respectively, while, at the depth of 20–40 cm, the mean values of exchangeable Mg in forest land, cultivated land, and grazing land were 5.47, 4.20, and 5.83 Cmol(+) kg^−1^, respectively. The highest and lowest values of exchangeable Mg were found under grazing land and cultivated land, respectively. The low exchangeable Ca and Mg under cultivated land might be due to leaching, soil erosion, and crop harvest as it was reported by [66].

##### 3.2.2.6. CEC

The ANOVA results (Table 4) revealed that the CEC of the soils was significantly (*p* < 0.05) affected by the land use types and the depth of the soil. The mean value of CEC under forest land, cultivated land, and grazing land at the depth of 0–20 cm was 37.55, 25.20, and 27.31 Cmol(+) kg^−1^, respectively. For the depth of 20–40 cm, the mean values of forest land, cultivated land, and grazing land were 24.10, 24.04, and 23.86 Cmol(+) kg^−1^, respectively (Table 4). This implies that the highest CEC value was found in the surface soil layer than in the subsurface. The significantly higher CEC in forest land might be due to the presence of higher SOM in the forest. This result is in line with the findings of [51] who reported that the CEC of soil was higher in forest land compared to that of the adjacent cultivated and grazing lands. This depletion of OC in farmland could be the result of intensive cultivation. These results were also in agreement with the findings of [66, 67].

The CEC values of the soil decreased with depth consistently from forest land to cultivated land. Similarly, Reference [68] reported that the CEC of soil was higher in the subsurface of the soil layer under the adjacent forest, cultivated, and grazing lands. But the result of the present study is unparalleled with the findings of [63] who reported that the CEC of the soil was not significantly affected by soil depth at the depth of 0–15 and 15–30 cm under adjacent maize, enset, and grasslands. The variation in the findings could be due to differences in perturbation factors [69].

## 4. Conclusion

Land use change is one of the important factors influencing the soil properties and exerts the most significant effects on the soil. Changes in land use and management can have profound effects on the quantity and dynamics of SOM and, in turn, on the soil ecosystem functions. SOC stocks and the physical and chemical properties of land uses in the study area were affected by land use systems and depth. SOC stock and most of the physical and chemical properties, including AvP, CEC, ex.K, and total N, were significantly higher in the forest compared to grazing land and cultivated land, while pH and BD were lower in forest land compared to cultivated and grazing lands. Thus, maintaining forest land, reducing the intensity of cultivation, and adopting integrated soil fertility management must be undertaken in the area for improving soil organic stock and soil properties.

## Figures and Tables

**Table 1:** Soil organic stock under the different land uses.

**Land use**	**BD**	**% SOC**	**SOC stock (ton ha** ^**−1**^ **)**
Soil depth (0–20 cm)			
Farm land	1.62^a^	1.33^a^	43.09^a^
Forest land	1.08^c^	3.79^c^	81.86^b^
Grazing land	1.63^a^	2.29^d^	57.71^c^
Soil depth (20–40 cm)			
Farm land	1.60^a^	1.19^b^	38.08^a^
Forest land	1.26^b^	2.79^d^	70.31^c^
Grazing land	1.31^b^	1.70^ab^	44.54^d^
*p*(0.05)	0.07	0.04	0.01

*Note:* Means with different letters between each land use indicate significant variation at 95% confidence interval.

**Table 2:** Textural class of the land uses.

**Land use**	**Depth (cm)**	**% sand**	**% silt**	**% clay**	**Textural class**
Cultivated land	0–20	61	33.5	5.5	Sandy clay loam
	20–40	58	30.5	11.5	Sandy loam
Grazing land	0–20	52	38	10	Sandy loam
	20–40	54	37	9	Sandy loam
Forest land	0–20	50	43.5	6.5	Loam soil
	20–40	45	44	11	Loam soil

**Table 3:** Effects of land use change on soil bulk density and soil moisture.

**Land uses**	**Depth (cm)**	**BD (g/cm** ^**3**^ **)**	**Moisture (%)**
Cultivated land	0–20	1.62^ab^	10.84^e^
	20–40	1.60^b^	10.86^e^
Grazing land	0–20	1.63^a^	14.76d
	20–40	1.31^c^	15.12^c^
Forest land	0–20	1.08^e^	20.6^b^
	20–40	1.26^d^	25.85^a^
*p*(0.05)		0.02	0.01

*Note:* Means with different letters between each land use indicate significant variation at 95% confidence interval.

**Table 4:** Effects of land use change on soil chemical properties.

**Land use**	**pH**	**%SOC**	**%TN**	**AvP**	**ex.Ca**	**ex.Mg**	**ex.K**	**CEC**
Soil depth (0–20 cm)								
Farm land	4.63^c^	1.33^e^	0.11^e^	12.63^c^	9.27^d^	5.83^c^	1.13^b^	25.20^b^
Forest land	6.02^a^	3.79^a^	0.35^a^	27.83^a^	23.30^a^	12.70^a^	1.51^a^	37.55^a^
Grazing land	5.14^b^	2.29^c^	0.20^c^	4.17^e^	14.43^b^	6.77^b^	1.04^b^	27.31^c^
Soil depth (20–40 cm)								
Farm land	5.20^b^	1.19^f^	0.10^f^	10.43^d^	6.87^e^	4.20^a^	0.88^cd^	24.04^b^
Forest	6.02^a^	3.10^a^	0.28^b^	24.52^b^	12.27^c^	6.17^b^	0.79^c^	24.10^c^
Grazing land	5.13^b^	1.70^d^	0.15^d^	2.43^f^	8.17^a^	5.47^a^	0.44^e^	23.86^b^
*p*(0.05)	0.03	0.05	0.01	0.05	0.02	0.04	0.016	0.045

*Note:* Means with different letters between each land use indicate significant variation at 95% confidence interval.

## Data Availability

Additional data may be available on request to the authors.

## Nomenclature

AvP: available phosphorus
ex.K, Mg, Ca: exchangeable potassium, magnesium, calcium, respectively
SOC: soil organic carbon
TN: total nitrogen
Ec: electrical conductivity
CEC: cation exchangeable capacity
